# The Diagnostic Accuracy of an Abbreviated vs. a Full MRI Breast Protocol in Detecting Breast Lobular Carcinoma: A Single-Center ROC Study

**DOI:** 10.3390/diagnostics15121497

**Published:** 2025-06-12

**Authors:** Francis Zarb, Deborah Mizzi, Paul Bezzina, Leanne Galea

**Affiliations:** Department of Radiography, Faculty of Health Sciences, University of Malta, MSD2080 Msida, Malta; deborah.mizzi@um.edu.mt (D.M.); paul.bezzina@um.edu.mt (P.B.); leanne.galea.15@um.edu.mt (L.G.)

**Keywords:** breast cancer, breast MRI, abbreviated breast MRI, receiver operator characteristics (ROC)

## Abstract

**Background/Objectives:** Abbreviated breast MRI protocols have been proposed as a faster and more cost-effective alternative to standard full protocols for breast cancer detection. This study aimed to compare the diagnostic accuracy of an abbreviated protocol with that of a full protocol in identifying lobular breast carcinoma using Breast Imaging Reporting and Data System (BI-RADS) classification. The diagnostic performance was evaluated against a gold standard comprising biopsy-proven lobular carcinoma or negative follow-up imaging, using Receiver Operating Characteristic (ROC) analysis and performance metrics such as sensitivity and specificity. **Methods:** A retrospective analysis was conducted on 35 breast MRI examinations performed between January 2019 and December 2021. Of these, 20 cases had biopsy-confirmed lobular carcinoma, and 15 were determined to be normal based on at least 12 months of negative follow-up imaging. Two radiologists independently reviewed the images using only the abbreviated protocol, blinded to the original reports. Their findings were then compared with the initial full-protocol MRI reports. BI-RADS categories 1 and 2 were considered negative for malignancy, while BI-RADS categories 3, 4, and 5 were considered positive. **Results:** The area under the ROC curve (AUC) was 1.0 for the full protocol and 0.920 and 0.922 for Radiologists A and B, respectively, using the abbreviated protocol. All malignant lesions were correctly identified by both radiologists across both protocols, resulting in a sensitivity of 100%. However, the abbreviated protocol demonstrated significantly lower specificity (73.3% for Radiologist A and 53.5% for Radiologist B) compared to 100% specificity with the full protocol (*p* < 0.05). Lymph node involvement was correctly identified in 6–7 of 7 cases, though Radiologist A reported four false positives. Lesion laterality and count matched histopathology in 75–90% of cancer cases depending on protocol. Lesion localization was accurate in 60–80% of cases using the abbreviated protocol, though size comparisons were limited due to the incomplete radiological documentation of dimensions. **Conclusions:** While the abbreviated MRI protocol achieved diagnostic accuracy and sensitivity comparably to the full protocol, it demonstrated reduced specificity. These findings suggest that abbreviated MRI breast protocol may be a viable screening tool, although the higher false-positive rate should be considered in clinical decision-making.

## 1. Introduction

Breast cancer remains the most frequently diagnosed cancer and the leading cause of cancer-related mortality among women worldwide [1]. Although all women are at risk, individual risk varies depending on the presence, type, and combination of several risk factors [2]. Designing breast screening programs that stratify women based on their specific risk profiles has the potential to improve early detection, particularly in younger age groups [1].

An effective screening test must meet several criteria: it should be safe, cost-effective, widely accessible, and capable of detecting disease with high sensitivity and specificity, ultimately leading to improved patient outcomes. Mammography is currently the standard modality for breast cancer screening due to its accessibility, cost-effectiveness, and suitability for population-level implementation [3]. However, its performance is significantly diminished in women with dense breast tissue and certain tumor growth patterns, which may obscure malignancies or mimic benign findings [4,5,6,7,8]. These challenges are even more pronounced in younger women and those with a high genetic risk, such as carriers of BRCA1 and BRCA2 mutations, whose screening often begins earlier in life [4,5,7,9]. For these individuals, mammography poses an additional concern due to their increased sensitivity to ionizing radiation.

Magnetic resonance imaging (MRI) is often recommended for high-risk women, as it avoids radiation exposure and provides superior soft tissue contrast. Despite its excellent diagnostic accuracy and sensitivity, independent of breast density, age, or tumor subtype, conventional breast MRI does not currently meet all the criteria for population-based screening. It is time-consuming, expensive, less accessible, and requires intravenous contrast administration, which may be a barrier in some settings [6,10,11,12,13].

To address these limitations, Kuhl et al. proposed an abbreviated MRI protocol in 2014, significantly reducing image acquisition and interpretation time while preserving diagnostic performance [14]. This innovation has generated growing interest in the potential use of an abbreviated MRI protocol for broader breast cancer screening applications, especially among high-risk populations. Several systematic reviews and meta-analyses have shown that abbreviated breast MRI protocol maintains high sensitivity (86–94.8%) and specificity (90–94.6%) compared to full-protocol MRI, though slightly lower sensitivity has been reported in some studies. Abbreviated breast MRI also improves workflow efficiency, with scan times reduced from 25 to 35 min to as little as 8 min and reading times averaging just over one minute. These advantages suggest the potential for increased throughput and reduced costs in high-demand settings [15,16,17].

Despite these strengths, abbreviated breast MRI has limitations. It may be less effective in characterizing lesions, particularly non-mass enhancements, due to the omission of sequences like T2-weighted imaging, potentially increasing recall rates and unnecessary biopsies. Additionally, the lack of standardized abbreviated breast MRI protocols across institutions hinders consistent implementation and comparative evaluation [18].

The purpose of this study was to assess the diagnostic accuracy of an abbreviated breast MRI protocol compared to the full MRI protocol in detecting lobular breast cancer, focusing on sensitivity, specificity, and clinical applicability.

This study focused specifically on invasive lobular carcinoma, a distinct histological subtype of breast cancer that accounts for approximately 10–15% of invasive cases. Invasive lobular carcinoma is known for its subtle and often inconspicuous appearance in mammography and ultrasound due to its diffuse, infiltrative growth pattern and minimal architectural distortion. These characteristics can lead to the underestimation of tumor size and extent, posing challenges for accurate staging and surgical planning. In contrast, MRI has demonstrated superior sensitivity in detecting invasive lobular carcinoma and in assessing multifocal or bilateral involvement [19]. Therefore, a targeted evaluation of MRI protocols in the context of invasive lobular carcinoma is clinically warranted and may inform imaging strategies tailored to this subtype.

## 2. Materials and Methods

This study employed a quantitative, retrospective, comparative, and non-experimental design. It was conducted at a public general hospital in a small European country with an approximate population of 500,000, where only about 150 breast MRI examinations are performed annually.

An image dataset was compiled from full-protocol MRI breast examinations performed between January 2019 (the earliest date breast MRI became available at the hospital) and December 2021. A retrospective consecutive sample was selected, consisting of patients who had undergone MRI scans using a 3.0 Tesla Philips Ingenia scanner with a dedicated 7-channel prone breast coil. All patients were scanned using the same full protocol (Table 1) and were included if they had either histopathological confirmation from a biopsy or a minimum of 12 months of negative follow-up imaging (mammography, US, or MRI) in the absence of biopsy. Findings representing the true disease status based on gold standard criteria were compared to the original full-protocol MRI report for patients with histopathologically confirmed lobular carcinoma.

Examinations were excluded if patients had breast implants or prior breast surgery as it was essential that breast tissue was fully visualized. Cases involving breast cancer subtypes other than lobular carcinoma were also excluded to ensure uniformity. Patient selection was performed using consecutive non-probability sampling, including all cases meeting the inclusion and exclusion criteria during the study period. This approach minimized sampling bias by incorporating the entire available population.

Sequences that comprised the abbreviated protocol were extracted from the full-protocol dataset (Table 1). The full MRI protocol for breast imaging included a series of standard and advanced sequences for comprehensive tissue characterization and lesion detection. These included axial T1-weighted turbo-spin-echo (TSE) and axial T2-weighted TSE sequences for baseline anatomical and fluid-sensitive imaging. Unenhanced axial dynamic THRIVE (T1-weighted with fat suppression) sequences were acquired prior to contrast administration to evaluate pre-contrast tissue signal. Post-contrast axial dynamic THRIVE sequences were obtained to assess enhancement kinetics. High-resolution sagittal eTHRIVE sequences were included for anatomical detail, and diffusion-weighted imaging (DWI) was performed to evaluate lesion cellularity and help differentiate between benign from malignant findings. This protocol reflects standard clinical practice and balances diagnostic accuracy with acquisition efficiency [20,21,22].

The abbreviated MRI protocol used in this study consisted of one unenhanced axial dynamic THRIVE (fat-suppressed T1-weighted) sequence and two post-contrast axial dynamic THRIVE acquisitions corresponding to the first and second early arterial phases following gadolinium administration. Unlike the protocol proposed by Kuhl et al. [14] which included one pre- and one post-contrast acquisition along with subtracted and maximum-intensity projection (MIP) images for interpretation, our abbreviated protocol did not include subtracted or MIP image reconstructions. Instead, interpretation was based on the direct evaluation of the dynamic THRIVE sequences reflecting local imaging practice to provide early kinetic information while maintaining a streamlined protocol.

The dataset included abbreviated-protocol MR images for 35 examinations, with 10 of these repeated to assess inter-rater reliability, resulting in a total of 45 examinations. Of the 35 cases, 15 were negative (normal) and 20 were positive (abnormal). Histology for the 20 abnormal cases confirmed invasive lobular carcinoma, all grade 2. Of these, 13 (65%) were in the left breast and 7 (35%) in the right. Lymph node involvement was present in seven (35%) of the cases. For the 15 negative cases, gold-standard confirmation was obtained via negative follow-up imaging for at least 12 months using mammography (n = 5), ultrasound (n = 5), MRI (n = 4), and DBT (n = 1), where ‘n’ refers to the number of cases.

The mean patient age was 57.9 years (range: 27–80 years). Clinical indications for MRI included breast cancer staging (n = 20, 57.1%), nipple discharge with a negative mammogram and ultrasound (n = 5, 14.3%), lesion characterization (n = 2, 5.7%), and screening due to family history or BRCA mutations (n = 8, 22.9%). These clinical indications were available to the radiologist when reporting the full-protocol MRI examinations.

During image evaluation, examinations were randomly presented to each radiologist. Two radiologists participated: one consultant breast specialist with over 10 years of experience and one resident breast specialist with over 5 years of experience. Although four radiologists were eligible to participate in this study, only two consented to take part. Each radiologist completed an image quality scoring sheet for each examination

The participating radiologists were blinded to patient history and had access only to the abbreviated-protocol image sequences, which were reviewed using their standard workstation through the Picture Archiving and Communication System (PACS). Workstations were equipped with monitors displaying 1280 × 1024 (pixels) and a maximum luminance of 200 cd/m^2^. Ambient illuminance, measured using a Physikalisch Technische Werkstatten CD LUX meter (model L991263), was 39 lux, within the range recommended by the American Association of Physicists in Medicine (report 270, 2019).

For each examination, radiologists first indicated whether a lesion was present. If one was present, further details were recorded: lesion size, location (breast side and quadrant), the number of lesions, lymph node involvement, and American College of Radiology (BI-RADS) 2013 classification. All lesions were categorized according to the BI-RADS classification system. A BI-RADS score of 1 or 2 was considered positive. Based on this classification threshold (BI-RADS ≥ 3), sensitivity, specificity, the positive predictive value (PPV), and the negative predictive value (NPV) were calculated.

Receiver Operator Characteristic (ROC) analysis was performed using SPSSv9 statistical software. ROC curves plot sensitivity (true positivity) on the y-axis against 1−specificity (false positivity) on the x-axis, representing diagnostic performance across varying thresholds. In this study separate ROC curves were generated for the abbreviated and full protocols. The Area Under the Curve (AUC) provides a measure of average diagnostic accuracy [23]. An AUC of 0.5 indicates no discrimination (random chance), while values between 0.5 and 0.7 indicate poor discrimination. AUCs between 0.7 and 0.8 reflect acceptable discrimination, values from 0.8 to 0.9 are considered excellent, and AUCs above 0.9 indicate outstanding diagnostic accuracy [24].

In addition to ROC analysis, several secondary imaging outcomes were assessed to evaluate the clinical adequacy of the abbreviated protocol beyond sensitivity and specificity. Lymph node involvement was analyzed based on the presence of histologically confirmed axillary metastasis, with MRI findings classified as suspicious or normal using established morphological criteria, such as cortical thickening and the loss of the fatty hilum. Lesion laterality and number were also recorded, with each radiologist noting the presence, side (left or right), and total number of suspicious lesions identified using both imaging protocols. These findings were compared with surgical and histopathological results. Lesion localization and size were assessed by documenting tumor position within the breast (quadrant level) and measuring lesion dimensions in the anteroposterior (AP), transverse (TRA), and craniocaudal (CC) planes, where available.

## 3. Results

### 3.1. Intra-Rater Reliability

Cohen’s Kappa test was conducted to evaluate intra-rater reliability for two binary variables, lesion presence and lymph node involvement. The Kappa values ranged from 0.6 to 0.78, indicating moderate to substantial agreement between radiologists. These findings suggest that the individual radiologists demonstrated consistent interpretations when reassessing the same cases.

For the BI-RADS scoring, the Intraclass Correlation Coefficient (ICC) ranged from 0.6 to 0.9, with *p*-values of 0.021 for Radiologist A and 0.0001 for Radiologist B, indicating statistically significant intra-rater agreement in both cases. This consistency further validates the reproducibility of BI-RADS assessments at the individual level.

### 3.2. Inter-Rater Reliability

Inter-rater agreement between the two radiologists was also assessed using Cohen’s Kappa. Results ranged from 0.6 to 0.8 with *p*-values < 0.05, indicating significantly moderate to substantial agreement between the radiologists.

The ICC for the inter-rater reliability of BI-RADS scores ranged from 0.4 to 0.8, with *p*-values of 0.086 and 0.0001. Although Radiologist B’s scores showed strong consistency, Radiologist A’s lower ICC weakened the overall agreement. Notably, Radiologist A consistently assigned lower BI-RADS scores than Radiologist B, suggesting a systematic difference in scoring behavior.

Clinically, these results highlight good agreement on binary variables (e.g., lesion presence, lymph node involvement) but variability in BI-RADS scoring. As BI-RADS classification influences patient management decisions, such inter-rater differences underscore the need for standardized training, calibration sessions, or decision-support tools, especially when using abbreviated MRI protocols.

### 3.3. ROC Curve and Diagnostic Accuracy

**Figure 1:**
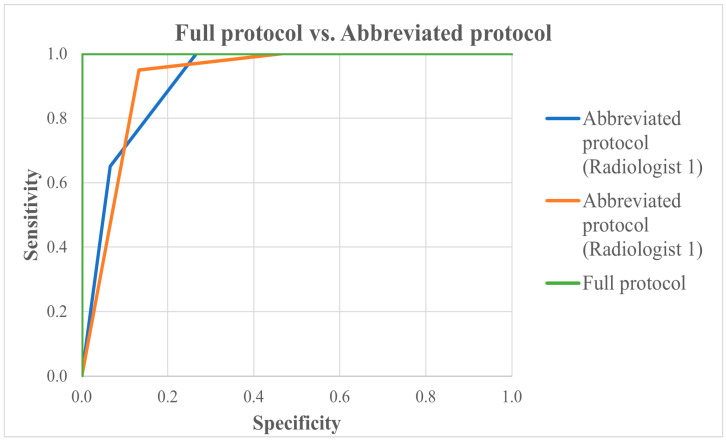
ROC—full vs. abbreviated protocols.

Receiver Operating Characteristic (ROC) curves were generated for both the full and abbreviated protocols (Figure 1), individually and combined for both radiologists. The gold standard was binary (1 = cancer present; 0 = cancer absent), while the diagnostic test values were based on the BI-RADS scores.

A comparison of Area Under the Curve (AUC) values using pairwise analysis yielded *p*-values > 0.05 (Table 2), indicating no statistically significant difference in diagnostic accuracy between the full and abbreviated protocols.

Each protocol demonstrated statistically significant diagnostic capability (*p* < 0.05; Table 3). The full protocol achieved an AUC of 1.0, while the abbreviated-protocol AUCs were 0.920 for Radiologist A and 0.922 for Radiologist B, all classified as “outstanding” in discriminative performance.

### 3.4. Full Protocol Analysis

The full protocol demonstrated perfect diagnostic performance, with an AUC of 1.0 and 100% accuracy across all performance indicators (Table 4), confirming its validity as the reference standard.

### 3.5. Abbreviated Protocol Analysis (Radiologist A—Consultant)

Radiologist A achieved an AUC of 0.92 with the abbreviated protocol, indicating outstanding discriminative ability. No cancer cases were missed. Overall diagnostic accuracy was 88.6% (31/35) (Table 5). Sensitivity, specificity, PPV, and NPV are summarized in Table 6.

### 3.6. Abbreviated Protocol Analysis (Radiologist B—Resident Specialist)

Radiologist B also achieved an AUC of 0.922. No cancer cases were missed. Diagnostic accuracy was 80% (28/35), indicating that the abbreviated protocol correctly classified 80% of cases (Table 6).

### 3.7. Lymph Node Involvement

Lymph node involvement was determined based on histologically confirmed axillary metastases aligned with morphological MRI criteria (e.g., cortical thickening, loss of fatty hilum) per Lee-Felker et al. [25]. Of the 20 breast cancer cases, 7 (35%) showed lymph node involvement.

**Figure 2:**
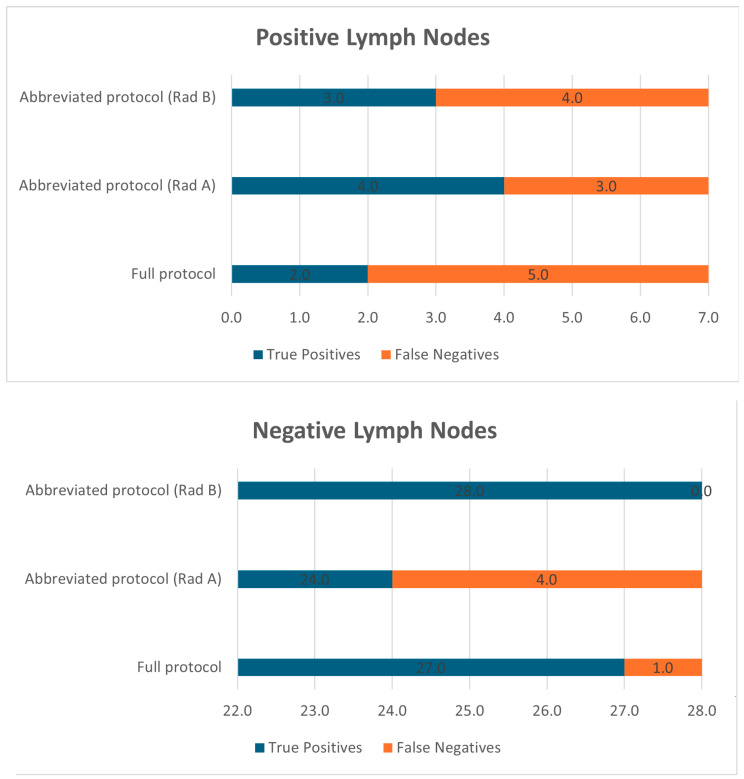
Lymph node involvement.

Radiologist A correctly identified all seven metastatic cases but reported four false positives. Radiologist B had one false negative but no false positives, demonstrating higher specificity. Performance comparisons are visualized in Figure 2.

### 3.8. Lesion Number and Laterality

Using the full protocol, both radiologists correctly identified lesion laterality in all cases. Using the abbreviated protocol, Radiologist A reported false positives in four negative cases (two right, two left), while Radiologist B reported seven (four right, three left). In one case, both misclassified the cancer as bilateral when it was unilateral (left).

For lesion count, full-protocol findings matched histopathology in 18 of 20 positive cases (90%). With the abbreviated protocol, both radiologists matched the lesion count in 15 of 20 positive cases (75%).

### 3.9. Lesion Size and Localisation

Lesion size comparisons were limited due to inconsistent reporting formats (e.g., incomplete use of standard orthogonal measurements: anteroposterior [AP], transverse [TRA], and craniocaudal [CC] axes). Furthermore, retrospective correlation with histopathology was constrained by incomplete surgical mapping.

Using the abbreviated protocol, Radiologist A correctly localized lesions by quadrant in 12 of 20 cases (60%), while Radiologist B correctly localized 16 of 20 cases (80%). Although lesion sizes were qualitatively comparable between protocols, quantitative comparison was not feasible due to inconsistent documentation.

## 4. Discussion

The abbreviated protocol demonstrated a sensitivity of 100% for both radiologists, matching the performance of the full protocol in detecting all biopsy-confirmed cancers. These findings align with previous studies by Heacock et al. (2015) and Mango et al. (2015) which also reported higher cancer detection rates using abbreviated protocols in cohorts with histologically confirmed malignancies [26,27]. Similarly, Petrillo et al. (2017) reported near-identical diagnostic performance between full and abbreviated protocols, detecting 206 out of 207 cancers [28]. Collectively, these findings reinforce the diagnostic utility of the abbreviated protocol for cancer detection. Despite high sensitivity, the abbreviated protocol yielded lower specificity compared to the full protocol, with values of 73.3% and 53.5% for Radiologists A and B, respectively. These results suggest an increased rate of false positives, a common limitation in abbreviated MRI studies [29]. False-positive results can lead to unnecessary follow-up procedures, including additional imaging, biopsies, and increased surveillance, all of which are associated with patient anxiety, health care costs and, in rare cases, procedural complications [29,30]. Furthermore, prior studies indicate that women undergoing biopsy after a false positive are significantly less likely to participate in future screening, with one study reporting a 2.3-fold reduction in follow-up screening attendance within 30 months [31].

Radiologist B’s lower specificity compared to Radiologist A’s may reflect differences in experience levels. The radiologic interpretation of breast MRI, particularly abbreviated protocols, demands a high degree of expertise, which may not parallel the learning curve of mammography [32]. Kuhl et al. (2014) emphasized the role of experience in reducing false-positive diagnosis, advocating for structured MRI training similar to that required in European mammography screening programs, which mandate the interpretation of 5000 cases annually [14]. These findings support the view that interpretive variability, rather than inherent technical limitations, may underlie many false positives in MRI. In clinical practice, this variability can compromise the specificity of abbreviated protocols when interpreted by less experienced radiologists. Therefore, the implementation of minimum case-volume requirements, dedicated fellowships, and standardized training may be necessary to improve MRI proficiency and reduce false-positive outcomes. Furthermore, the standardization of both acquisition and interpretation criteria is critical to enhancing diagnostic consistency and reproducibility across different readers. Although the abbreviated protocol successfully identified all cases of lobular breast cancer in this study, false positives were still present in 11.4% (four) and 20% (seven) of normal cases for Radiologists A and B, respectively. These results are higher than those reported by Oldrini et al., (2018) [33] who found false-positives rates of 4.9% (7) and 8.4% (12) among senior (5 years’ experience) and junior (6 months’ experience) radiologists, albeit in a larger sample of 90 women. Across other studies [28,34,35], the specificity of the abbreviated protocol has consistently been lower than that of the full protocol. The current study’s relatively low specificity (<90% as reported in other studies [19,36]) may partly be attributed to the lack of access to prior imaging and biopsy results. This limitation hindered the radiologists’ ability to assess lesion stability over time or use other imaging modalities for comparison. A comparable study [37] also reported similarly low specificity (45% and 52%) under the same constraint of limited prior data.

Notably, the NPV of the abbreviated protocol was 100% for both radiologists, equivalent to that of the full protocol and consistent with the previous literature [14,28,34,35,38]. The high NPV underscores the protocol’s strong ability to confidently exclude malignancy. In contrast, the PPV, which inversely relates to false-positive rates [39], was lower for the abbreviated protocol, at 83.3% for Radiologist A and 74% for Radiologist B, compared to 100% for the full protocol. These figures are consistent with Chen et al. (2017) [34] who also found a reduced PPV in abbreviated protocols (22%) compared to full protocols (40%). A lower PPV, while not affecting sensitivity, indicates a greater burden of unnecessary follow-up procedures.

### Study Limitations

This study has several limitations. First, the sample size was small (n = 35). This single-center study limited the number of patients meeting the inclusion criteria, i.e., a small sample size (n = 35) was used for image evaluation, which limits the generalizability of findings and reduces statistical power. While the paired design improved internal validity by controlling for inter-patient variability, the limited number of both positive and negative cases raises the risk of Type II error and wide confidence intervals around sensitivity and specificity estimates. These results should be considered exploratory and generate a hypothesis applicable only to the specific cohort studied. Larger, multi-center trials are needed to confirm and expand upon these findings.

Second, only two radiologists participated, each with differing levels of experience. This limits the ability to generalize results across broader clinical settings and introduces interpretive variability. Future studies should include a larger and more diverse group of readers to assess the reproducibility of abbreviated MRI protocols across experience levels.Third study focused on lobular breast cancer, limiting its applicability to other breast cancer subtypes. Although lobular carcinomas are known to pose diagnostic challenges, especially in mammography, this focus restricts the broader relevance of findings.

## 5. Conclusions

Based on the findings of this study, and supported by the current literature, several key recommendations can be made to enhance the clinical utility and implementation of abbreviated breast MRI protocols.

Radiologist expertise plays a pivotal role in the accurate interpretation of breast MRI. It is recommended that minimum experience thresholds are established similar to mammographic screening programs that mandate a specified number of cases annually. The development of dedicated training programs and fellowships focusing on breast MRI, particularly on abbreviated protocols, is essential to ensure radiologist competency. Continuing professional development (CPD) and ongoing education should also be prioritized to maintain interpretive accuracy and reduce inter-observer variability.

The standardization of abbreviated breast MRI protocols is equally critical. Institutions should adopt uniform imaging sequences and standardized interpretation frameworks, such as BI-RADS for MRI, to ensure consistency across clinical practice and research. The development of international consensus guidelines will further support standardized implementation and facilitate cross-study comparability.

To maintain high diagnostic performance, quality assurance measures should be integrated into breast MRI screening workflows. The regular auditing of radiologist performance metrics, such as sensitivity, specificity, and recall rates, along with the peer review of imaging interpretations, can help identify discrepancies and promote continuous improvement. Benchmarking outcomes against full-protocol MRI and mammography can provide valuable clinical context.

From a clinical perspective, abbreviated breast MRI should be considered a viable screening tool for specific high-risk populations including BRCA mutation carriers and women with dense breast tissue. It may also serve as a suitable alternative for women unable or unwilling to undergo full-protocol MRI. Incorporating abbreviated breast MRI into risk-stratified screening strategy models can optimize diagnostic benefit while managing resource utilization.

Operationally, the reduced acquisition and interpretation time of abbreviated MRI improves patient throughput and lowers associated costs, making it particularly advantageous in high-volume or resource-constrained settings. However, clinicians must remain aware of its limitations, particularly the exclusion of sequences like T2-weighted imaging which may impair lesion characterization. In such cases, referral for full-protocol MRI or expert review should be considered to minimize unnecessary recalls or biopsies.

Finally, further research is needed to support broader adoption. Large-scale, prospective multi-center trials are required to validate long-term diagnostic performance. Policy efforts should aim to incorporate abbreviated breast MRI into national breast screening strategies where appropriate. Additionally the potential role of artificial intelligence (AI) as a decision-support tool warrants exploration, particularly to support less experienced readers and enhance diagnostic consistency.

## Figures and Tables

**Table 1:** Standard full-protocol and abbreviated-protocol sequences.

Standard Full-Protocol Sequences	Scan Time (Minutes)	Abbreviated-Protocol Sequences (Extracted from Standard-Protocol Examinations)	Scan Time (Minutes)
Axial T1-Weighted TSE	5.41	/	-
Axial T2-Weighted SPAIR	6.10	/	-
Unenhanced Axial Dynamic THRIVE (T1-Weighted) (FS)	1.18	Unenhanced Axial Dynamic THRIVE (T1-Weighted) (FS)	1.18
Ninth Contrast Axial Enhanced Dynamic THRIVE (T1-Weighted) (FS)	9.14 total	First and Second Contrast Axial Enhanced (Early-Arterial-Phase) Dynamic THRIVE (T1-Weighted) (FS)	3.54 total
eTHRIVE High-Resolution Sagittal	4.21	/	-
Diffusion-Weighted Imaging (DWI)	3.27	/	-
T2-Weighted Long TE		/	
High-Resolution Axial	4.02	/	-
Maximum-Intensity Projection (MIP)	0	Maximum-Intensity Projection (MIP)	0
Subtraction Imaging	0	Subtraction Imaging	0
Total Scan Time	33.33		4.72

**Table 2:** Results from the pairwise comparison of each protocol.

Protocol	AUC	*p*-Value (Asymptotic Sig.)	Asymptotic 95% Confidence Interval	
			Lower Bound	Upper Bound
Full protocol	1.00	0.00	1.00	1.00
Abbreviated protocol (Radiologist A)	0.92	0.00	0.82	1.00
Abbreviated protocol (Radiologist B)	0.92	0.00	0.81	1.00

**Table 3:** Resultant AUCs and *p*-values from ROC curves for both full and abbreviated protocols.

Protocol	Asymptotic		AUC Difference	Asymptotic 95% Confidence Interval	
	z	Sig. (2-Tail)		Lower Bound	Upper Bound
Full protocol vs. Abbreviated protocol A	1.65	0.10	0.08	−0.02	0.18
Full protocol vs. Abbreviated protocol B	1.62	0.11	0.08	−0.02	0.17
Abbreviated protocol A vs. Abbreviated protocol B	−0.04	0.97	−0.00	−0.09	0.09

**Table 4:** Performance indicators for the full protocol.

Performance Indicators	Value
Sensitivity	100%
Specificity	100%
PPV	100%
NPV	100%

**Table 5:** Image ratings by radiologists.

True Disease Status	1 Negative	2 Benign	3 Probably Benign	4 Suspicious	5 Highly Suggestive of Malignancy	6 Malignancy (Biopsy-Proven)
Radiologist A: Normal	4	7	1	2	1	0
Radiologist A: Abnormal	0	0	1	6	13	0
Radiologist B: Normal	8	0	2	3	2	0
Radiologist B: Abnormal	0	0	0	1	19	0

**Table 6:** Performance indicators for the abbreviated protocol for both radiologists.

Performance Indicators	Value (Radiologist A)	Value (Radiologist B)
Sensitivity	100.%	100%
Specificity	73.3%	53.3%
PPV	83.3%	74%
NPV	100%	100%

## Data Availability

The data presented in this study are available on request from the corresponding author due to ethical reasons.

## Abbreviations

The following abbreviations are used in this manuscript:

AUC	Area Under the Curve
BI-RADS	Breast Imaging Reporting and Data System
BRCA	Breast Cancer Gene
DBT	Digital Breast Tomosynthesis
DWI	Diffusion-Weighted Imaging
FREC	Faculty Research Ethics Committee
ICC	Intraclass Correlation Coefficient
MIP	Maximum-Intensity Projection
MRI	Magnetic Resonance Imaging
NPV	Negative Predictive Value
PACS	Picture Archiving and Communication System
PPV	Positive Predictive Value
ROC	Receiver Operating Characteristics
SPSS	Statistical Package for Social Sciences Software
T	Tesla
UREC	University Research Ethics Committee
US	Ultrasound
